# Supplementary material for: Low Bone Mineral Density and Risk for Osteoporotic Fractures in Patients with Chronic Pancreatitis
Source: Nutrients. 2021 Jul 13;13(7):2386. doi: 10.3390/nu13072386 (PMC8308495; doi:10.3390/nu13072386)
Supplement: Supplementary file 1 [file nutrients-13-02386-s001.zip › nutrients-1291401-supplementary.pdf]

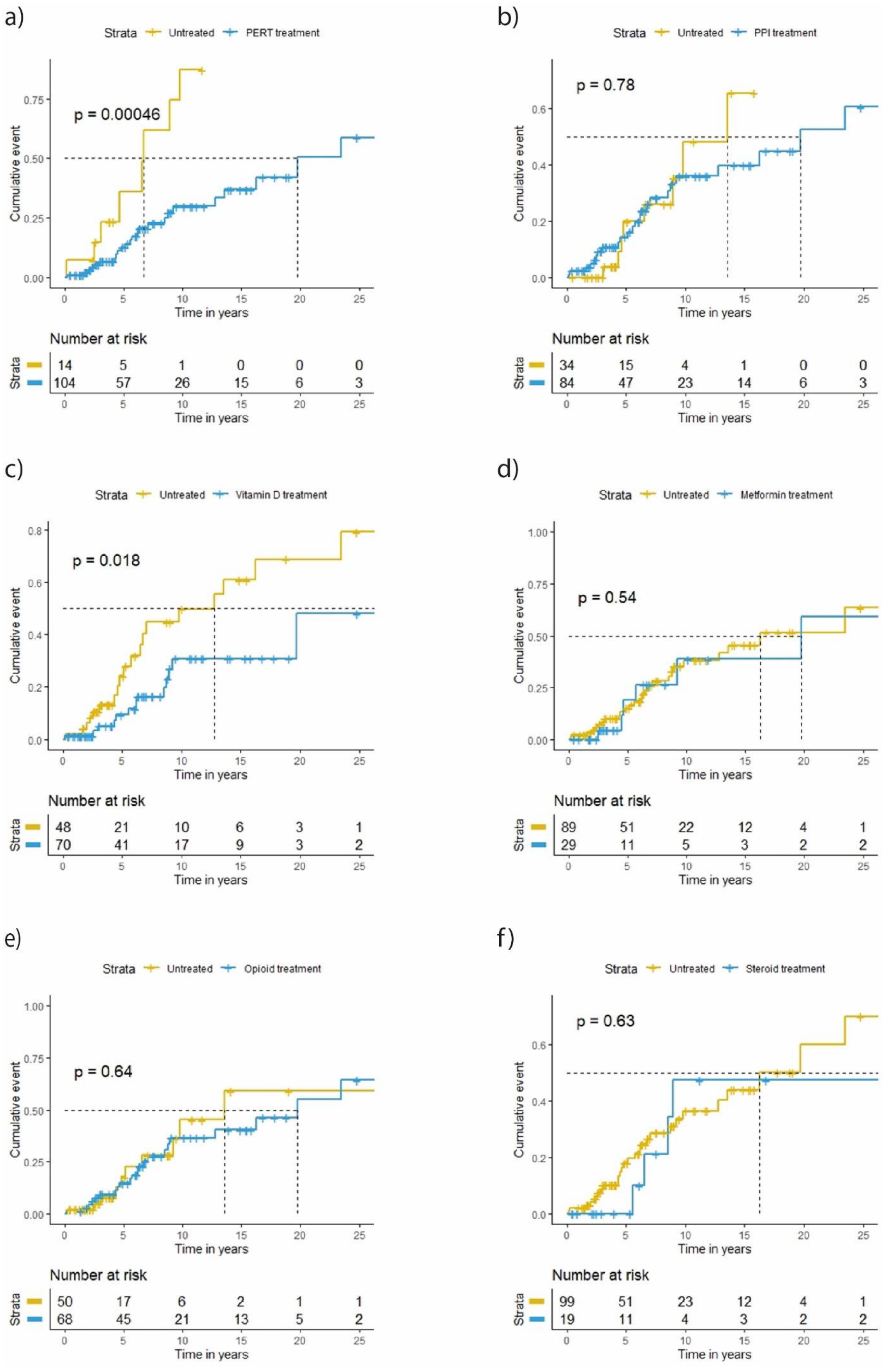
a)
Strata Untreated PERT treatment
p = 0.00046
Cumulative event
Time in years
Number at risk
14 5 1 0 0 0
104 57 26 15 6 3
b)
Strata Untreated PPI treatment
p = 0.78
Cumulative event
Time in years
Number at risk
34 15 4 1 0 0
84 47 23 14 6 3
c)
Strata Untreated Vitamin D treatment
p = 0.018
Cumulative event
Time in years
Number at risk
48 21 10 6 3 1
70 41 17 9 3 2
d)
Strata Untreated Metformin treatment
p = 0.54
Cumulative event
Time in years
Number at risk
89 51 22 12 4 1
29 11 5 3 2 2
e)
Strata Untreated Opioid treatment
p = 0.64
Cumulative event
Time in years
Number at risk
50 17 6 2 1 1
68 45 21 13 5 2
f)
Strata Untreated Steroid treatment
p = 0.63
Cumulative event
Time in years
Number at risk
99 51 23 12 4 1
19 11 4 3 2 2

Figure S1. Kaplan-Maier curves of cumulative fracture events for all patients stratified by **a)** PERT supplementation, **b)** PPI treatment, **c)** Vitamin D supplementation, **d)** Metformin treatment, **e)** Opioid treatment and **f)** Steroid treatment. Treatment or supplementation was defined as 90 or more consecutive days of regular treatment at any time before the fracture event (PERT=pancreatic enzyme replacement therapy; PPI=proton pump inhibitor).

Table S1. Cox regression analysis adjusted hazard ratio (aHR) of fracture events in patients treated with PERT, PPI, Vitamin D, Metformin, Opioids or Steroids during 90 or more consecutive daily regular treatments at any time before the fracture event.

Treatment	aHR* [95% CI]
PERT	0.3 [0.1,0.7]
PPI	1.2 [0.5,2.7]
Vitamin D	0.4 [0.2,0.8]
Metformin	0.8 [0.3,2.2]
Opioids	1.2 [0.6,2.6]
Steroid	0.6 [0.2,1.5]

aHR was adjusted for sex, age and BMI.
